# Spectrum of Large and Medium Vessel Vasculitis in Adults: Primary Vasculitides, Arthritides, Connective Tissue, and Fibroinflammatory Diseases

**DOI:** 10.1007/s11926-022-01086-2

**Published:** 2022-09-27

**Authors:** Luca Seitz, Pascal Seitz, Roxana Pop, Fabian Lötscher

**Affiliations:** 1 Department of Rheumatology and Immunology, Inselspital, University Hospital, University of Bern, Freiburgstrasse, CH-3010 Bern, Switzerland; 2 Immunodeficiency Laboratory, Department of Biomedicine, University Hospital and University of Basel, Basel, Switzerland; 3 Department of Infectious Diseases and Hospital Hygiene, University Hospital, University of Zurich, Zurich, Switzerland

**Keywords:** Vasculitis, Aortitis, Differential diagnosis, Connective tissue disease, Fibroinflammatory disease, Arthritis

## Abstract

**Purpose of Review:**

To provide a comprehensive overview of the spectrum of large and medium vessel vasculitis in adults with primary vasculitides, arthritides, connective tissue, and fibroinflammatory diseases as well as vasculitis mimics, for an efficient differential diagnosis and initial diagnostic approach.

**Recent Findings:**

Imaging has had a tremendous impact on the diagnosis of medium to large vessel vasculitis, now often replacing histopathologic confirmation and identifying new disease manifestations (e.g., intracranial disease in giant cell arteritis; vascular manifestations of IgG4-related disease). Novel diseases or syndromes involving blood vessels have been described (e.g., VEXAS-Syndrome with polychondritis). The use of the terms “medium” or “large” vessel varies considerably between medical specialties.

**Summary:**

The differential diagnosis of large and medium vessel vasculitis is becoming increasingly complex as new entities or disease manifestations of known inflammatory rheumatic diseases are regularly identified. A more precise and widely recognized definition of the vessel sizes would make future research more comparable.

## Introduction

This review is intended as a comprehensive guide for the differential diagnosis of suspected medium to large vessel vasculitis (MVV, LVV) in adults. Primary vasculitides and vasculitis in arthritides, connective tissue, and fibroinflammatory diseases are discussed. MVV and LVV in the context of primary immunodeficiency, autoinflammatory, neoplastic, or infectious diseases, as well as drug induced vasculitis, are considered elsewhere [1••]. The complex topic of single organ vasculitis (SOV) was recently reviewed in this journal [2]. The first part of the article is dedicated to a discussion of the difficulties in classifying vasculitis by vessel size (Table 1). The individual diseases associated with MVV and LVV are discussed subsequently. Thereafter, a brief overview of vasculitis mimics is provided, as consideration of these disorders is essential in the diagnostic process. We only consider diseases without inflammation of the vessel wall as vasculitis mimics (Table 2). Table 3 provides additional details and pearls important for diagnosis of individual diseases causing MVV and LVV.

**Table 1:** Variable definitions of vessel sizes

	Small vessels	Medium vessels	Large vessels
Chapel Hill 2012 nomenclature [3]^(i)^	Intraparenchymal arteries, arterioles, capillaries, venules, and veins	Main visceral arteries and veins and their initial branches	Aorta and its major branches and analogous veins
Cardiovascular pathology viewpoint [4]	Arterioles, capillaries, venules	Small- and medium-sized arteries throughout the body (including both distributing and intraparenchymal arteries)	Aorta and aortic arch branches, distributing arteries of the extremities and neck
Dermatopathology viewpoint [5•]	Arterioles, capillaries, post-capillary venules (found both in the dermis and subcutis)	Small arteries or small veins (diameter <800 μm, four to eight medial muscular layers without distinct tunica adventitia) found in the subcutis or dermal-subcutis junction	(not present in skin)
Neuroradiology viewpoint [6]^(ii)^	Cerebral arteries with lumen diameter < 0.75 mm (e.g., lenticulostriate artery)	Cerebral arteries with lumen diameters of 0.75–2.0 mm (M3/4-, A2-5, and P2-5- segments of middle, anterior and posterior cerebral arteries; posterior inferior, anterior inferior and superior cerebellar arteries; M2- and A1/P1-segments are usually also considered medium sized)	Internal carotid, vertebral and basilar artery, M1-segment of middle cerebral artery
Daily practice (rheumatology) ^(iii)^	Arterioles, capillaries, venules, small intraparenchymatous Arteries and veins (e.g., retinal arteries with diameter of approximately 0.15 mm)	Remaining muscular arteries and corresponding veins (including splanchnic and renal vessels)	All elastic arteries and “large” distributing muscular arteries and corresponding veins (aorta, main pulmonary, Brachiocephalic, common/internal carotid, vertebral, subclavian, axillary, brachial, common and external/internal iliac, femoral, popliteal)

(i) For vessel size classification, the text refers to a figure intended to represent medium and large vessels; however, it remains unclear which vessels are effectively classified as large or medium. (ii) In neuroradiology, the definitions of vessel sizes are influenced by thrombectomy trials (imaging with digital subtraction angiography); the use of 7-Tesla MRI will probably influence these definitions in the future, as the image resolution is constantly improving. (iii) Because the diameters of basilar, renal, cephalic, common hepatic and the proximal mesenteric arteries are often similar to the smaller vessels of the “large artery” category (i.e., brachial, popliteal, internal iliac, vertebral), this classification remains arbitrary

**Table 2:** Mimics of medium or large vessel vasculitis

Mainly arterial dilatation or aneurysm formation	Mainly arterial stenosis or occlusion	Potentially mixed presentation: Dilatation/aneurysm formation and/or stenosis/occlusion
Marfan’s syndrome (LV/MV)	Transient perivascular inflammation of the carotid artery syndrome (LV) (i)	Atherosclerosis (LV/MV)
Ehlers-Danlos syndrome (LV/MV)	Embolic diseases (e.g., cholesterol emboli, cardiac myxoma) (MV)	Radiation-induced vasculopathy (LV/MV)
Loeys-Dietz syndrome (LV/MV)	Sickle cell disease (MV)	Erdheim-Chester disease (LV/MV)
Osteogenesis imperfecta (LV/MV)	Hypercoagulable states (including livedoid vasculopathy, cryofibrinogenemia, thrombotic microangiopathies, or antiphospholipid syndrome) (MV)	Hypereosinophilic syndromes (LV/MV)
Familial non-syndromic aortic aneurysm and dissection syndrome (LV)	Drug-induced occlusion, esp. digital artery (cocaine/amphetamine, chemotherapy etc.) (MV)	Segmental arterial mediolysis (MV)
	Calciphylaxis (MV)	Neurofibromatosis (LV/MV)
	Primary hyperoxaluria (MV)	Fibromuscular dysplasia (LV/MV)
	Primary amyloidosis (MV)	Cutis laxa (LV/MV)
	Malignancy of the vessel wall (e.g., aortic intimal sarcoma) (LV)	Fabry disease (LV/MV)
	Malignant infiltration of the vessel wall (LV/MV)	Hyperhomocysteinemia (LV/MV)
	Intravascular lymphoma (MV)	Direct mechanic trauma to vessel (LV/MV) (e.g., Hypothenar-hammer syndrome)
	Pseudoxanthoma elasticum (LV/MV)	Dissection/intramural hematoma (LV/MV)
	Moyamoya disease/syndrome (LV/MV)	Periarterial inflammation (e.g., pancreatitis, infection) (LV/MV)
	Reversible cerebral vasoconstriction syndrome (MV)	Post-stenotic aneurysms (MV)
	Cerebral vasospasm after trauma/SAH (MV)	
	Mid-aortic syndrome (LV)	
	Coarctation of the aorta (LV)	
	Congenital arterial hypoplasia (e.g., vertebral artery) or stenosis (e.g., pulmonary artery) (LV/MV)	
	Thoracic outlet syndrome (LV)	

(i) Classification as a vasculitis mimic is controversial because of the presence of inflammatory wall infiltrates. *MV* medium vessels, *LV* large vessels, *SAH* subarachnoid hemorrhage

**Table 3:** Primary vasculitides, arthritides, connective tissue, and fibroinflammatory diseases causing medium and large vessel vasculitis in adults

Category/disease	Epidemiology, patient characteristics	Mainly affected large and medium vessels or vessel beds ^(i)^		Clinical manifestations ^(ii)^	Associated laboratory findings	Diagnostic pearls and pitfalls ^(iii)^
		Large	Medium			
Primary vasculitides						
Giant cell arteritis	Usually > 50 (> 40) Y/A; f > m (3:1); ↑ Northern European ancestry and northern latitude; common (incidence ~10/10^5/y in population >50 Y/A) [7]	‡ Aorta and all direct large branches, supraaortal (subclavian, axillary, brachial) > lower extremities (iliac, femoral, popliteal) [8]	‡ Head/neck (any artery, esp. TA, facial, occipital, ophthalmic) +/- Mesenteric, renal, lateral/internal thoracic, infrabrachial, infrapopliteal, coronary, cerebral, ovarian, uterine, breast, skin, liver, testes [9–13••]	Headache, scalp tenderness, abnormal TA (palpation), polymyalgia, jaw/limb claudication, arterial bruits, decreased pulse, blood pressure-difference, visual loss, double vision, fever, weight loss, dry cough, vertigo/hearing loss, stroke, pericarditis, mononeuritis, plexopathy [14•]	CRP or ESR ↑ > 95%, rarely both →; thrombocytosis (usually < 800 G/L), mild anemia ~ 50%, mild leukocytosis ~ 20%; liver enzymes 2–4× ↑ (GGT/AP > AST/ALT) ~ 20% [15•, 16]	• Imaging findings of TA do not allow differentiation between different types of vasculitides • Screen for GCA in the context of pathological vessel wall imaging of the proximal intracranial arteries • Think of GCA with CRP ↑ and unclear liver enzyme elevation, dry cough, pericarditis, or fever of unknown origin
Takayasu Arteritis	Usually < 40 Y/A (< 60); f > m (3:1); ↑ Japan, West Asia (similar worldwide); very rare (incidence ~ 1–2/10^6/y); potentially associated: SPA/IBD [7, 17, 18]	‡ Aorta, subclavian, CCA, ICA, vertebral, axillary, brachial, iliac, femoral +/- pulmonary, popliteal [19, 20••]	‡ renal, mesenteric +/- coronary, celiac, proximal intracranial, ophthalmic [20••–22]	Fatigue, arthralgia, myalgia, fever, arterial bruits, reduced pulse, blood pressure difference, limb claudication, carotidodynia, headache, hypertension, dizziness, syncope, stroke, mesenteric ischemia, uveitis/scleritis, myocardial infarction, skin lesions [11 ,19]	CRP or ESR ↑ ~ 70% at initial diagnosis; mild anemia ~ 30%; other findings (mild thrombocytosis or leukocytosis, creatinine ↑ in renal artery stenosis) [19]	• Normal ESR/CRP does not exclude TAK • Screen for IBD (fecal calprotectin) and assess IgG/A/M levels (common variable immunodeficiency?) [1••] • Typical angiographic patterns: involvement of the abdominal vessels, symmetric aortic arch disease, or focal disease [20••]
Polyarteritis nodosa **(**classic**)**	Usually 40–60 Y/A (childhood to senescence); m > f (~ 1.5:1); ↑ Caucasians (classical type); very rare (incidence: classical type ~ 1/10^6/y, any type ~ 1–8/10^6/y in Europe) [7]	+/- ICA, vertebral, (very rare: femoral, aorta (vasa vasorum)) [23–26]	‡ Muscle, skin (incl. digital arteries), renal, any gastrointestinal (mesenteric, hepatic, splenic etc.), peripheral nerves, testicular +/- TA, coronary, ocular, ovary, uterus, cerebral [25, 27]	Weight loss, myalgias, arthralgia, fever, hypertension mononeuritis multiplex, livedo/ulcers/nodules, scant purpura, renal/bowel infarction or bleeding, orchitis, multifocal myositis, myocarditis, pericarditis, pancreatitis, stroke, subarachnoid hemorrhage (aneurysms), pachymeningitis, retinal vasculitis, scleritis, uveitis, keratitis, pleural effusion [28–30]	CRP ↑ and /or ESR >30 mm/1h in ~ >75% (normal esp. in localized forms); non-glomerular hematuria/proteinuria; mild to moderate eosinophilia and anemia; creatinine ↑; AST/ALT →/↑; ANCA and cryoglobulins →; C3/4 →/↑; CK rarely ↑ [28, 29]	• Esp. in young adults, consider testing for ADA2-deficiency • If histology shows affection of capillaries or venules, PAN is not the likely diagnosis (arterioles are rarely affected) • Digital subtraction angiography remains the most sensitive imaging for microaneurysms and medium vessel vasculitis • Deep excisional biopsy of a non-ulcerated skin nodule, a combined nerve/muscle or muscle biopsy are ideal specimens
Kawasaki disease	Usually young children, very rarely 18–50 Y/A; f = m; ↑ Japan and East Asia (rest of world much rarer); incidence in adults unknown [7, 31•]	‡ aorta, CCA, subclavian, axillary, brachial, iliac, pulmonary [32, 33]	‡ Coronary, renal, mesenteric, hepatic, splenic +/- lower leg, cerebral, testicular, muscle [32, 34, 35]	Fever, unspecific rash, conjunctival injection, cheilitis, strawberry tongue, erythema of hands/feet, periungual desquamation, lymphadenopathy, arthralgia; chest pain (coronary aneurysm, myocarditis, pericarditis, pleuritis); hepatitis (jaundice), pancreatitis, colitis, stroke, aseptic meningitis, cranial nerve palsy [36]	CRP ↑↑, rarely → with late presentations; common (leukocytosis, thrombocytosis, hyperferritinemia, moderate liver enzyme ↑); occasional (eosinophilia, sterile leukocyturia) [31•, 37]	• Differential diagnosis: infections (esp. Epstein-Barr-virus, parvovirus, human immunodeficiency virus, Streptococcus, SARS-Cov2), consider causes of coronary aneurysms [10, 31•] • SARS-Cov2-induced multisystem inflammatory syndrome can result in a Kawasaki-like presentation in young adults [1••]
ANCA-associated vasculitis	Usually > 40–50 Y/A (rare in children, more common with increasing age); f ≈ m; incidence all subtypes (~ 25/10^6/y), EGPA ~ 1–4/10^6 [7]	+/- aorta (incl. periaortitis), ICA [38–40]	+/- TA (symptoms can be like GCA), muscle, coronary, cerebral, ocular, hepatic, mesenteric, gastric (splanchnic vessel rarely with aneurysms and rupture) [41••–44]	MPA/GPA/EGPA: constitutional symptoms, arthralgia, lung fibrosis or hemorrhage, GN, purpura, neuropathy, peri-myocarditis, bowel ischemia/ulcers, polychondritis, digital gangrene, meningitis, stroke, scleritis, retinitis, keratitis, rhinosinusitis, sialadenitis. GPA: mass lesions, nasal septal perforation, otitis, subglottic stenosis, pachymeningitis. EGPA: asthma (>95%). [45, 46]	CRP ↑ (rarely → in localized forms); creatinine ↑, active sediment (GN); IgG4 often ↑; eosinophilia frequent (EGPA ↑↑); PR3- or MPO-ANCA ↑ >85% in GPA/MPA (→ in localized forms); EGPA: ANCA ↑ ~ 35% (MPO >> PR3); IgE/ECP ↑ [47]	• In high probability situations with negative ANCA, a repeat immunoassay using a different platform should be performed • Eosinophilic pleural and pericardial effusions occur in EGPA • ANCA is not 100% specific: e.g., a patient with supraaortic large artery vasculitis with low-titer ANCA still more likely has GCA • Because IgG4 elevation is common in AAV, it should always be included in its differential diagnosis.
Cryoglobulinemic vasculitis	Usually 40–70 Y/A (childhood to senescence); f > m (~ 2:1); rare disease (unknown incidence); primary/secondary ~ 20/80% (infections, autoimmune diseases (esp. Sjogren’s, SLE, RA), lymphoproliferation) [48•, 49]	+/- aorta (vasa vasorum, very rare) [50, 51]	+/- “PAN-like” distribution (especially skin, nerve, muscle, gastrointestinal) [49, 52–54]	Palpable purpura, livedo, ulcers/necrosis of skin, digital gangrene, Raynaud’s, arthralgia, arthritis, GN, polyneuropathy, mononeuritis multiplex, stroke, cerebral vasculitis (small vessels), bowel ischemia, myelitis, pulmonary hemorrhage [48•, 49]	CRP/ESR ↑ (can be →); monoclonal or mixed cryoglobulins detectable; mild anemia; C4 ↓ ~ 70-90%; RF often ↑↑ (rarely →); active urinary sediment (GN); serum immunofixation (paraprotein?) [55, 56]	• Immunoglobulins can be spuriously low due to cryoglobulins • Always extensively screen for secondary causes in CV • Direct immunofluorescence of acute skin lesions often reveals deposits of IgM > IgG > IgA, and/or complement • CV can mimic IgA-vasculitis (usually monoclonal IgA) • Cryoglobulins are common in hepatitis C without CV
Cogan’s syndrome	Usually 18–40 Y/A (described 2–81y); f = m; worldwide (most cases published Caucasian); very rare (unknown incidence); potential association with IBD [57–59•]	‡ Aorta (esp. ascending), CCA, subclavian; +/- Vertebral, axillary, iliac, femoral, popliteal [58•]	+/- Tibial, peroneal, renal, splenic, mesenteric, coronary [58•, 60]	Interstitial keratitis, Menière-like symptoms, scleritis, uveitis, episcleritis; in minority of cases: fever, weight loss, fatigue, arthralgia/myalgia, testicular pain, unspecific rash, splenomegaly, pericarditis, pleuritis, Raynaud’s; headache, aseptic meningitis, encephalitis, cranial/peripheral neuropathy [57, 58•]	CRP or ESR ↑ ~ 75–90%; leukocytosis ~ 75% (rarely > 20 × 10^9/L), mild anemia/thrombocytosis ~ 30%; rare: mild eosinophilia, low titers of RF or ANA [57, 58•, 61]	• In patients without the classical symptom combination (interstitial keratitis and Menière-like symptoms), the risk for misdiagnosis is high and the important differential diagnoses should be considered thoroughly: BS, polychondritis, IBD, sarcoidosis, Susac syndrome, Vogt-Koyanagi-Harada disease, AAV, infections (Whipple, Lyme, Chlamydia)
Behçet’s syndrome	Usually onset 2nd to 3rd decade (occurs from childhood to late adulthood); m = f; prevalence variable (high in regions with ↑ HLA-B51 positivity - Southwest Asia, Mediterranean region); vascular disease ~ 5–40% of all BS (↑ young males) [62•]	Veins (thrombosis): ‡ lower extremity veins; +/- vena cava, subclavian Arteries (aneurysms): ‡ Pulmonary (typically with aneurysms), abdominal aorta, CCA, iliac, femoral, popliteal; +/-Subclavian, axillary, brachial. [62•, 63]	Veins: ‡ Every size and location possible (also superficial thrombophlebitis) +/- Cerebral veins/dural sinuses Arteries: +/- Mesenteric, renal, splenic, coronary aneurysms (all rare) [62•, 63]	General: bipolar aphthosis, uveitis (typically bilateral, anterior- to panuveitis), retinal vasculitis, cutaneous (papulopustulosis, erythema nodosum), pathergy (specific), abdominal pain, arthralgia and arthritis, CNS-symptoms (parenchymatous or vascular disease). Vascular disease associated with: constitutional symptoms, venous thrombosis, Budd-Chiari syndrome, CVST with seizures and cerebral hemorrhage, hemoptysis due to pulmonary artery aneurysms. [62•, 63]	No specific histologic or laboratory findings. CRP and ESR usually ↑; calprotectin ↑ in gastrointestinal disease; HLA-B51 positive ~ 50–60% (of limited aid in diagnosis)	• Vascular manifestations typically occur within first 5 years • Hughes-Stovin syndrome should be evaluated as BS • Thrombosis commonly occurs without embolism • Cardiac involvement is rare but possible (intracardiac thrombosis, coronary aneurysms, endocarditis) • Repeated occlusion of vascular stents, despite proper anticoagulation, should lead to consideration of BS
Connective tissue diseases						
Systemic lupus erythematosus	Usually in child-bearing age (occasionally > 50 Y/A); mean age in vasculitis ~ 38 y; f >> m; worldwide (non-Caucasians ↑); incidence SLE ~ 1-25/10^5/y; any type of vasculitis in ~ 11–35% of SLE [64, 65]	+/- Aorta, iliac (occasional “TAK-like” distribution supraaortal and iliac/femoral) [66–68]	+/- “PAN-like” (skin, mesenteric, coronary, peripheral nerves, gallbladder, kidney, pancreas, muscle), cerebral [64, 69–72]	Vasculitis: Skin (palpable purpura, livedo, panniculitis, ulcers, digital ischemia, urticaria); gastrointestinal (abdominal pain, mesenteritis, enteritis, pancreatitis, cholecystitis, bowel ischemia/necrosis and hemorrhage of small > large bowel, pancreatitis); heart (myocardial infarction, aneurysms); nervous system (mononeuritis, mononeuritis multiplex, stroke, seizures) [64, 69]	CRP →/ ↑, ESR ↑; cytopenias of all cell lineages common; C3/4 →/↓; creatinine ↑, proteinuria, active sediment (GN); IgG →/ ↑; ANA ↑; antibodies →/ ↑ (SSA/B, Sm, RNP, dsDNS, histone, C1q, RF, nucleosomes, APS-antibodies) [64]	• Vasculitis usually coincides with highly active phases of SLE, and can be the presenting feature in < 20% • Screening for cryoglobulins should always be performed • Non-vasculitic vasculitis mimics need to be considered in SLE, particularly in the CNS (sequelae of APS or Libman-Sacks endocarditis, accelerated atherosclerosis and thrombocytopenic thrombotic purpura)
Sjogren’s syndrome	Usually 30–60 Y/A (occurs from childhood to senescence); f >> m (~ 10:1); worldwide; incidence of SS ~ 7/10^5/y; any type of vasculitis in 5–32% of SS [73]	Questionable association: periaortitis, aortitis [74•, 75]	+/- “PAN-like,” without aneurysms (skin, peripheral nerves, gastrointestinal, kidney, salivary glands, muscle); unclear if true vasculitis in CNS [76–78]	General: dry eyes/mouth (dysphagia), fatigue, fever, arthralgia, myalgia. Vasculitis: purpura, urticaria-like lesions, skin ulcers; mononeuritis multiplex; mesenteritis, bowel infarction; hematuria. (for manifestations of CV, see above) [76–78]	CRP →/ ↑, ESR ↑; anemia, thrombocytopenia, leukopenia all possible; immunofixation of serum (possible paraprotein); IgG →/ ↑; ANA →/ ↑; antibodies →/ ↑ (SSA/B, RF); cryoglobulins →/ ↑; C4 ↓ in CV [74•]	• In SS with vasculitis, cryoglobulins need to be screened for • Non-vasculitic manifestations in the peripheral nervous system (neuronopathy, demyelinating polyneuropathy, small fiber neuropathy) must be differentiated from vasculitic neuropathy • AAV can rarely co-exist with SS: ANCA-testing is advised • Visceral angiography is expected to be normal
Idiopathic inflammatory myopathies	Usually 40–60 Y/A (occurs from childhood to senescence); f > m (~ 2:1); worldwide; incidence any IM ~ 1–19/10^6/y; any type of vasculitis in ~ 9-30% of IM (DM > other IM) [73]	(iv)	+/- Gastrointestinal (DM), cerebral (anti-synthetase syndrome, DM), pulmonary, skin [73, 79–81]	General: fatigue, weight-loss, myalgia, weakness, dysphagia, skin findings (Gottron’s sign and papules, heliotrope rash, and many others), arthralgia/arthritis, Raynaud’s, calcinosis, interstitial lung disease Vasculitis: skin purpura, livedo, ischemic/hemorrhagic stroke, intestinal ischemia/perforation. [73, 79–81]	CRP/ESR →/ ↑; CK (→)/↑-↑↑, troponin-T often ↑ (Troponin-I usually →); AST/ALT often ↑ (pseudo-hepatic); ANA →/ ↑ (nuclear/cytosolic); myositis antibodies ↑ ~ 60-80% (usually a panel is ordered) [82]	• In suspected IM, capillaroscopy should be considered • A negative result of immunofluorescence (HEp-2 cells, nuclear or cytoplasmatic), does not rule out myositis antibodies • Vasculitis of muscle arteries can result in multifocal myositis and normal to elevated CK (esp. AAV, PAN) [83]
Relapsing polychondritis	Usually 30–60 Y/A (occurs from childhood to senescence); m = f; worldwide; very rare, incidence ~ 0.7–3.5/10^6/y [84]	‡ Aorta (thoracic > abdominal) +/- Innominate, subclavian, CCA, ICA, vertebral, iliac, axillary, femoral [85, 86•]	+/- Coronary (most frequently affected medium-sized artery), renal, celiac, mesenteric, hepatic, cerebral [85, 86•]	Constitutional symptoms, chondritis (auricular ~ 80%, nasal ~ 40% (saddle nose), bronchial/larynx 50% (hoarseness, stridor, cough), joints ~ 40% (polyarthralgia/-itis), costal chondritis), ocular ~ 45% (episcleritis, scleritis, uveitis), ear ~ 27% (sensorineural hearing loss, vertigo), aortic insufficiency (dilatation of ascending aorta), skin (purpura, aphthous ulcers), aseptic meningitis, encephalitis, stroke [84, 87]	No specific laboratory findings; CRP ↑ ~ 90%; anemia and thrombocytosis frequent, leukocytosis and eosinophilia occasional; cytopenias possible (associated with myelodysplastic syndrome) [84]	• In any patient presenting with RP, an extensive laboratory screening for secondary causes (esp. systemic vasculitides, RA, SLE, SS, or myelodysplasia) should be performed • Pulmonary function tests and eye examination should be performed to screen for possible bronchial or ocular disease • In males with RP: if MCV is > 100 fl and a platelet count of < 200×10^3/μl is found, it may be VEXAS-syndrome [88••]
Arthritides						
Rheumatoid vasculitis	Usually onset > 60 Y/A, median 10–15y after RA diagnosis; m > f; incidence RV ~3.9/10^6/y; risk factors: smoking, severe RA (erosive, nodulosis, extraarticular manifestations), also in “burnt out” RA [89–91]	+/- Aorta (esp. ascending); questionable association: “TAK-like” (rare cases) [89, 92••, 93]	‡ “PAN-like” (usually without microaneurysm): skin, muscle kidneys, peripheral nerve, gastrointestinal (mesenteric, splenic, pancreatic, hepatic), +/- Testes, rarely coronary and cerebral [89–91]	General: Constitutional symptoms, myalgia, arthritis, nodulosis. Vasculitis: skin ~ 90% (nail fold infarcts, livedo, scant palpable purpura, ulcers, and digital gangrene); mononeuritis multiplex 40%; eye (small vessels: scleritis, ulcerative keratitis, retinal vasculitis); other (alveolar hemorrhage, bowel ischemia, testicular or myocardial infarction, pancreatitis, stroke) [89–91]	CRP/ESR ↑; anemia/thrombocytosis frequent; C3/4 →/ ↓; RF and/or anti- cyclic-citrullinated peptide typically ↑↑; p-ANCA without subspecificity and cryoglobulins are occasionally detected [89, 90]	• Inactive RA with extraarticular activity at RV onset common [89] • Peripheral ulcerative keratitis points to imminent systemic RV • A combined skin and muscle biopsy increases diagnostic yield • Histology typically shows granulomatous inflammation in large- (sometimes rheumatoid nodules of aortic wall), pauciimmune fibrinoid necrosis in medium- and leucocytoclasia with immune complex deposition in small-vessel vasculitis [92••]
Spondyloarthritis	Usually adolescence to 40 Y/A (exceptionally > 60y); m > f (~ 2-3:1); worldwide (↑ with high HLA-B27 prevalence); prevalence of SPA ~ 0.2–1.6%; vasculitis rare, ↑ in longstanding disease and HLA-B27 positivity [94]	‡ aorta (root and ascending >> descending aorta); unclear association: periaortitis [94]	(iv)	General: arthralgia/arthritis, inflammatory back pain, enthesitis, uveitis, symptoms of IBD Vasculitis: usually asymptomatic; dilatation of aortic root and/or ascending aorta can result in secondary aortic valve insufficiency, fibrosis can result in cardiac conduction disorders (atrioventricular block typical) [94, 95•]	CRP/ESR often →, can be ↑; HLA-B27 positive in majority (negative result does not rule out SPA); mild anemia common	• If pure aortic regurgitation develops in SPA, consider screening for aortitis (FDG-PET/CT recommended) • If dilatation of the aortic root and/or ascending aorta is found in patients without other identifiable risk factors or family history, occult SPA may be present
Fibro-inflammatory diseases						
IgG4-related disease	Mean age of onset ~ 57 Y/A (occurs from childhood to senescence); m > f (~ 3:2); worldwide; rare disease (incidence unknown) [96, 97]	‡ Aorta (aortitis ~8%, thoracic aorta ↑; RPF or periaortitis ~17%, abdominal aorta ↑), iliac +/- CCA, vertebral, subclavian, pulmonary, vena cava [97–99]	+/- Coronary, TA, mesenteric, splenic, celiac, renal, cerebral, skin (formation of aneurysms is common in medium sized arteries) [99, 100]	General: often mild symptoms, weight loss, fatigue, arthralgia, atopy. Any organ can be affected by infiltration/mass lesions: single organ ~25%, ⩾ 2 organs ~75%, on average 3 organs. Frequently affected: pancreas/liver/bile ducts, salivary glands, orbit, dura mater, aorta, and retroperitoneum (abdominal pain, hydronephrosis, aneurysm and rupture) [96]	CRP/ESR often →, can be ↑ (~ 30%); eosinophilia (~ 30%); IgE ↑ (~ 60%), IgG →/ ↑; IgG4 ↑ (~ 60–70%, in aortitis more common than periaortitis); C3/4 often ↓; ANA ↑ ~ 30%; plasmablasts ↑ in peripheral blood common [101, 102•]	• IgG4 ↑ is not specific, consider: AAV, SLE, SS, RA, GCA, liver cirrhosis, Erdheim-Chester disease etc. [102•] • Ultrasound of the salivary glands should be performed; they can be biopsied easily and involvement is frequent • Lymph node biopsy is usually unspecific and not diagnostic
Chronic periaortitis	Usually 40– 70 Y/A (occurs from early adulthood to senescence); m > f (~ 2–3:1); idiopathic form incidence ~ 1-10/10^6/y; risk factors: smoking, asbestos exposure [103•]	‡ Abdominal aorta (usually infrarenal), iliac +/- Thoracic aorta, subclavian, innominate, proximal CCA [103•, 104]	+/- renal, celiac, mesenteric [103•, 104]	General: insidious onset of low-grade fever, fatigue, and weight loss; pain ~ 90% (abdomen, lumbar or inguinal regions, flanks). Ureteral obstruction with hydronephrosis (~ 60–70%), renal atrophy/failure. Scrotal or inguinal pain, hydrocele, varicocele. Venous encasement: leg edema/thrombosis. Rare: renal hypertension, laryngeal nerve palsy/cough, limb or abdominal claudication [103•]	No specific laboratory findings. CRP and/or ESR ↑ ~ 80%; anemia of chronic disease frequent; unspecific ANA ↑; IgG4 →/↑; creatinine ↑ (postrenal obstruction); anti-thyreoperoxidase ↑ (Hashimoto thyroiditis associated with CP) [103•]	• Serum IgG4 is often normal in biopsy-proven IgG4-RPF [103•] • Before diagnosing an idiopathic form of CP, it is advisable to consider and screen for the many secondary forms • GCA and other types of aortitis may cause some periarterial fat-stranding on CT, but not actual RPF; luminal narrowing does not usually occur in CP (but it can in TAK > GCA)
Miscellaneous						
Sarcoidosis	Usually 30– 60 Y/A (occurs from childhood to senescence); f ≈ m; African American > Caucasian; incidence of vasculitis unknown, but rare; vasculitis can occur any time during disease course [105]	+/- Aorta and direct large branches (“TAK-like”), main pulmonary artery and veins [105–107]	+/- Mesenteric, hepatic, celiac, splenic, renal, coronary, TA, medium sized pulmonary arteries and veins, cerebral arteries/veins [105, 106, 108, 109•]	General: fever, sarcoid manifestations in other organs (most common: pulmonary, skin, eyes, and liver) Vasculitis: small vessels: skin, peripheral nerves, lung; “PAN-like”: abdominal pain; large vessels: pulse differences/claudication, carotidodynia, pulmonary hypertension; CNS: lacunar/hemorrhagic strokes, rarely territorial infarction or CSVT [105, 106, 109•, 110]	CRP ↑ ~ 50%; serum/urinary calcium often ↑; 1,25-Vitamin-D3 →/ ↑; angiotensin converting enzyme and soluble IL2-Receptor ↑ ~ 50%; IgG ↑ common (polyclonal); liver enzymes, troponin or creatinine ↑ in respective organ manifestations [111]	• To identify sarcoidosis, examination of eyes, scars and tattoos and screening for hypercalcemia/hypercalciuria are helpful • An extracerebral biopsy target and large vessel vasculitis can most efficiently be identified with FDG-PET-CT • Serum Ig levels should be ordered (common variable immunodeficiency disorders can result in granulomatous disease with “TAK-like” presentation) [1••]
Thrombangiitis obliterans	Usually onset ~ 30–35 Y/A (range 20 - 50 Y/A); m > f (~ 5:1); Asia ↑ (especially West and East Asia); prevalence is declining; > 95 % are smokers [112, 113•]	(iv)	‡ Infrabrachial and infrapopliteal arteries, superficial veins +/- Splanchnic, coronary, (rarely described TA, cerebral, kidneys, testes) [112, 114–116]	Claudication of limbs, ischemic ulcers and gangrene, migratory thrombophlebitis (~ 60%), Raynaud’s (not symmetrical), mild sensory abnormalities, migratory arthralgia or mild arthritis (~ 10%, wrist and knees most common, usually in pre-occlusive phase) [112, 113•, 116]	No specific laboratory findings. CRP is almost always → (rarely mildly ↑); autoantibodies and cryoglobulins are not detectable. [112, 113•]	• Non-vasculitic occlusive vasculopathy should be excluded (i.e., cryofibrinogenemia, APS, thrombophilia, embolic states etc.) • Thrombangiitis obliterans should be diagnosed cautiously in non-classical locations lacking histological confirmation, since other vasculitides and mimics can look very similar on imaging.

(i) Main vessels or vessel beds affected as described in the literature (i.e., arteries or vessel beds not listed, may still be affected); if not specified otherwise, the vessel names indicate arteries. (ii) A selection of important and pertinent clinical manifestations is provided; the list is not exhaustive; the order is not according to frequency. (iii) Pearls mostly reflect the personal experience of the authors and are therefore only partially referenced. (iv) Not identified by our literature search. AAV, ANCA-associated vasculitis; ADA2, adenosine deaminase 2; ANA, anti-nuclear antibody; ANCA, anti-neutrophil cytoplasmic antibodies; ALT, alanine aminotransferase; AP, alkaline phosphatase; APS, antiphospholipid syndrome; AST, aspartate aminotransferase; BS, Behçet syndrome; CCA, common carotid artery; CK, creatine kinase; CNS, central nervous system; CRP, c-reactive protein; CV, cryoglobulinemic vasculitis; CVST, cerebral venous sinus thrombosis; DM, dermatomyositis; ECP, eosinophil cationic protein; ESR, erythrocyte sedimentation rate; EGPA, eosinophilic granulomatosis with polyangiitis; FDG-PET/CT, fluorodeoxyglucose-positron emission tomography/computed tomography; GCA; giant cell arteritis; GGT, gamma-glutamyltransferase; GN, glomerulonephritis; GPA, granulomatosis with polyangiitis; HLA, human leukocyte antigen; ICA, internal carotid artery; IBD, inflammatory bowel disease; Ig, immunoglobulin; IM, idiopathic inflammatory myopathies; MPA, microscopic polyangiitis; PAN, polyarteritis nodosa; RA, rheumatoid arthritis; RF, rheumatoid factor; RP, relapsing polychondritis; RPF, retroperitoneal fibrosis; RV, rheumatoid vasculitis; SLE, systemic lupus erythematosus; SPA, spondyloarthritis; SS, Sjogren’s syndrome; TA, temporal artery; TAK, Takayasu arteritis; VEXAS, (vacuoles, E1 enzyme, X-linked, autoinflammatory, somatic); Y/A, years of age

^‡^Typically affected vessels or vessel beds

^+/-^Occasionally to rarely affected vessels or vessel beds

^↑^Elevated/more frequent

^↑↑^Markedly elevated

^→^No change/normal/negative

^↓^Depressed/below normal

^~^Approximately

Traditionally, vasculitides are grouped according to the affected vessel size (small, medium, and large). This can be helpful in approaching the diagnosis in an individual patient. The allocation of a disease to the categories LVV, MVV, and small vessel vasculitis (SVV) is based on the *typical* vessel size affected. In reality, many diseases can affect vessels of any size, and even typical LVVs often involve primarily medium-sized vessels [3]. The division of vessels into these categories seems simple at first glance, but the terms are used very inconsistently in the literature, e.g., a small artery in neuroradiology would be defined as a medium-sized artery in dermatology. The presence of a vasa vasorum vasculitis (a SVV) in the wall of larger vessels (MVV or LVV) even further complicates the topic. The definitions are strongly influenced by different diagnostic approaches: in dermatology by histology, in neuroradiology by imaging, in other fields by a combination of these approaches [3–6]. Because there is no universal classification system for vessel size, it is understandable that different medical specialties approach this classification in the way that best serves their clinical practice. This makes the comparison of publications challenging. Table 1 briefly compares examples of published categorizations by authors of a selection of different specialties and shows the differing definitions. Because Chapel Hill’s nomenclature remains ambiguous, for this review we have classified vessels according to our daily rheumatological practice following the Chapel Hill nomenclature, but also attempting to define large vessels more clearly: distributing vessels proximal to the elbow, knee, and dura mater are included in this category [3].

## The Primary Vasculitides

Most cases of MVV and LVV will fall into this category. Due to the tendency to use imaging for diagnostic confirmation, other types of vasculitides and mimics should be carefully considered in every patient.

### Giant Cell Arteritis

Giant cell arteries (GCA) is the most frequent disease causing MVV or LVV. Apart from classic polymyalgic and cranial symptoms, GCA should be considered in a variety of other presentations, including patients with constitutional symptoms with elevated C-reactive protein (CRP) and/or erythrocyte sedimentation rate [14•]. Most arteries, especially any elastic large and large medium-sized arteries above the clavicles, but not capillaries, venules, or arterioles, can be affected [8–12]. Since SVV is found in the eye (e.g., cilioretinal artery) and in temporal artery (TA) biopsies, the possibility of SVV should be considered in other organs [117]. If the typical histology is found unexpectedly in resected organs, e.g., uterus, a SOV must be considered. In postulated isolated aortitis, GCA needs to be screened for thoroughly [2]. While GCA was assumed not to cause central nervous system (CNS) vasculitis, vessel-wall MRI changed that paradigm; usually it represents extension of extradural disease and involves the proximal intracranial arteries [13••].

### Takayasu Arteritis

Especially outside Asia, Takayasu arteries (TAK) is an extremely rare disease of mostly young women [7]. Symptoms are often vague, but characteristic findings such as arterial bruits aid in its identification [19]. Several diseases have been found to coexist with TAK or to cause “TAK-like” LVV (e.g., common variable immunodeficiency, systemic lupus erythematosus (SLE), inflammatory bowel disease, spondyloarthritis, sarcoidosis) [17, 18]. Whether it is a co-occurrence of two diseases or just one disease with a “TAK-like” appearance is a difficult question and subject of debate, especially for spondyloarthritis and inflammatory bowel disease a co-occurrence seems to be likely [1••]. TAK causes mainly LVV and MVV of the larger medium-sized arteries, including the CNS [19, 21, 22]. Because retinal and skin vasculitis can occur, rare SVV manifestations should be considered [11, 118].

### Polyarteritis Nodosa

A severe, necrotizing vasculitis, with propensity for aneurysms of visceral medium-sized arteries [28]. The definition of polyarteritis nodosa (PAN) has changed considerably in the past, and by definition neither capillaries nor venules are involved [3, 119••]. While classic PAN is the idiopathic form, in secondary or non-classical PAN, another disease manifests in a “PAN-like” fashion: infections (e.g., HBV, HCV or HIV), autoinflammatory or immunodeficiency diseases (e.g., familial Mediterranean fever; adenosine-deaminase-2-deficiency), blood dyscrasias (e.g., hairy-cell leukemia, myelodysplastic and myeloproliferative syndromes), and systemic rheumatic diseases [1••, 28, 119••]. Therefore, in the case of a necrotizing ANCA-negative MVV, both "PANlike" disorders and mimics (e.g., segmental arterial mediolysis) need to be differentiated. The clinical manifestations of classical PAN range from severe systemic to localized presentations, where differentiation of systemic PAN from SOV is necessary (e.g., cutaneous-limited forms or isolated involvement of testicles) [29, 120]. Pulmonary capillaritis is not compatible with PAN and prominent CNS affection, usually a rare and late feature, should trigger consideration of adenosine deaminase-2 deficiency [1••, 23, 30]. Any small- to medium-sized artery can potentially be affected; LVV is extremely uncommon [24–27].

### Kawasaki Disease

A typical childhood disease that can also occur in young adults [31•]. The presentation is similar to children, with dreaded cardiac (esp. coronary aneurysm) and neurological sequelae [36]. Being so rare in adults, infectious mimics of its clinical presentation (fever, rash etc.) and other diseases causing coronary aneurysms need to be evaluated [1••, 10, 31•, 36]. Apart from the coronaries, involvement of other large and distributing medium-sized arteries has been described in autopsies of children. A similar distribution of vessel inflammation is expected in adults, but data are limited [32–35].

### Anti-neutrophil Cytoplasmic Antibody(ANCA)-Associated Vasculitis

The anti-neutrophil cytoplasmic antibody-associated vasculitis (AAV) are subdivided into granulomatosis with polyangiitis (GPA), microscopic polyangiitis (MPA), and eosinophilic granulomatosis with polyangiitis (EGPA), with EGPA set apart in terms of frequency (very rare) and clinical presentation [3]. There is no “usual” presentation, any organ can be affected, and myriads of symptoms and signs are possible. EGPA almost exclusively presents in patients with asthma and the ANCA-negative subtype is difficult to differentiate from other eosinophilic diseases [45, 121]. It is unclear whether necrotizing vasculitis occurs in hypereosinophilic syndrome or whether it is just an anti-neutrophil cytoplasmic antibody (ANCA)-negative EGPA. MPA and GPA partially overlap in clinical presentation, but only the later has a tendency for destructive mass lesions [46]. Most manifestations of AAV are due to SVV, and MVV/LVV are very rare (approximately 1–5%) [38–40, 41••, 42]. Cerebral MVV was occasionally described [43].

### Immune Complex Vasculitides

Typically, immune complexes cause SVV. For urticaria vasculitis or anti-glomerular basement membrane disease, no cases with MVV or LVV could be identified by our literature search. For the few cases of IgA-vasculitis with possible MVV or LVV, a definite etiological link was not demonstrated [122]. **Cryoglobulinemic Vasculitis(CV)**: CV is predominantly secondary to infections (esp. HCV), lymphoproliferative or autoimmune diseases [48•]. Manifestations of occlusive vasculopathy (Raynaud’s or cold-induced acral necrosis) can occur with high, especially monoclonal, cryoglobulin levels. CV usually presents as SVV; rarely and mainly in HCV-positive patients, it can additionally cause “PAN-like” necrotizing MVV [5•, 49, 52, 53]. A single case with a clinical presentation like cranial GCA was identified; histology showed an adventitial SVV with an otherwise unremarkable TA [54]. LVV is a rarity as only two cases with HCV infection could be identified (histologically a vasa vasorum vasculitis) [50, 51].

### Cogan’s Syndrome

A very rare disease without a clear definition [57, 58•]. The diagnosis requires the presence of eye inflammation and inner-ear dysfunction, classically interstitial keratitis and Menière-like symptoms [57]. In the majority of patients, either ocular or vestibulocochlear symptoms are initially present and within approximately 3–6 months the other manifestation occurs [58•]. The division into “typical” (interstitial keratitis and Menière-like symptoms occurring within *less* than 2 years) and “atypical” (other ocular inflammation and Menière-like symptoms occurring within *less* than 2 years or interstitial keratitis and Menière-like symptoms occurring *more* than 2 years apart) CS is mostly historical [123]. Vasculitis can be identified in about 20% of patients and can affect any vessel size. SVV of the inner ear, eyes (retinal vasculitis), and skin was described [124]. The typical manifestation is a MVV to LVV with similar manifestations like in PAN or TAK, respectively [58•, 60]. TA affection was not identified by our search. Whether intracranial arteries are affected remains unclear [125].

### Behçet’s Syndrome

Behcet’s syndrome (BS) is of unknown etiology. Its manifold clinical manifestations include the affection of arteries and veins of all sizes in 5–40%; thus, BS is regarded as a “variable vessel vasculitis” [3, 62•]. Certain features of BS, i.e., the response to colchicine or the recurrent and self-limiting nature of some manifestations, imply a polygenic autoinflammatory pathogenesis [126]. The major symptom of BS is the presence of recurrent oral ulcerations; while this symptom alone is non-specific, its absence makes the diagnosis of BS unlikely. Young men are generally more severely affected than women and more commonly show vascular manifestations. Affected vessels have a strong tendency for thrombosis, but the risk of embolism is low due to the strong wall adherence of thrombi. Selected thrombotic manifestations in BS (e.g., Budd-Chiari syndrome and aneurysms of the pulmonary arteries, including Hughes-Stovin syndrome) are associated with a high mortality rate [63].

## Vasculitis in Connective Tissue Diseases and Arthritis

In general, SVV is more common in these disorders, but MVV and LVV do occur [73]. Vasculitis usually manifests in patients with established disease and is rarely one of the initial manifestations. In systemic sclerosis, vasculopathy does not reveal the histological characteristics of vasculitis. The numerous published cases of MVV and LVV tend to represent overlapping diseases (esp. AAV or CV) rather than true systemic sclerosis associated vasculitis [73]. In mixed connective tissue disease, SVV of the retina, bowel, or skin can occur and a vasculopathy like in systemic sclerosis can affect the arteries of the distal extremities. No definite case of MVV or LVV could be identified, but in overlapping syndromes with other connective tissue disease, more diverse vasculitic presentations are expected.

### Systemic Lupus Erythematosus

Lupus vasculitis rarely manifests as MVV or LVV, the most common presentation is SVV affecting the skin in 90% of cases (usually leukocytoclastic). Less frequently, also the lungs, liver, pancreas, bowel, and nervous system can be involved [64, 69, 70, 73]. Lupus vasculitis rarely presents in a “PAN-like” fashion with necrotizing vasculitis in multiple organs, predominantly of small arteries and occasionally with MVV and development of aneurysms [64, 69]. Especially mesenteric vasculitis is characteristic for SLE with conventional angiography often showing normal findings but occasionally demonstrating typical findings of MVV with aneurysms. Endoscopic biopsies usually do not show the culprit vessel [71, 72]. LVV is very rare; histopathology reveals lymphoplasmacytic infiltration of the adventitia and media or vasa vasorum vasculitis with fibrinoid necrosis. In the occasionally described “TAK-like” disease in patients with SLE, it usually remains unclear whether it is a manifestation of SLE or two different diseases (i.e., TAK and SLE concomitantly). A case report revealing histologic evidence of vasa vasorum vasculitis of an iliac artery suggests that SLE can cause LVV with a “TAK-like” morphology without TAK being present concomitantly [66–68, 92••].

### Sjogren’s Syndrome

Vasculitis usually occurs as a late manifestation in Sjogren’s (median 10 years after diagnosis), with SVV presentation in >95% of cases (usually leukocytoclastic, occasionally lymphocytic) and MVV in <5%, which might be underreported and usually coexists with SVV of the skin [76, 77, 74•]. Since cryoglobulins are common in Sjogren’s, CV should always be considered [74•]. Rarely, a “PAN-like” necrotizing vasculitis of small medium-sized arteries (without aneurysms) occurs in internal organs [74•, 77]. Rarely, based solely on angiographic findings, MVV of the CNS has been reported, but a definite etiologic association of these findings with Sjogren’s syndrome was not proven [78]. Periaortitis or aortitis, diagnosed by FDG-PET, was also described in a few patients, also without a clear direct etiological link to the primary disease [74•, 75].

### Idiopathic Inflammatory Myopathies

Apart from SVV of the skin and muscle in dermatomyositis (DM), inflammatory myopathies only rarely present with vasculitis. In DM, especially juvenile DM, a severe gastrointestinal vasculitis with possible MVV was described [73]. Apart from pulmonary capillaritis, a rare MVV of pulmonary arteries was demonstrated in “polymyositis” in older autopsy cases [79]. In anti-PL-12-anti-synthetase-syndrome and in DM, MVV of the CNS was demonstrated (in angiographically MVV, histologically a SVV was detected) [80, 81]. Cases with LVV could not be identified.

### Relapsing Polychondritis

Relapsing polychondritis (RP) primarily affects cartilaginous and proteoglycan-rich (eyes, blood vessel, or inner ears etc.) tissues and is either idiopathic or secondary to systemic diseases (approximately 30%). Since secondary RP is frequently due to primary vasculitides, the clear differentiation to vasculitis as a manifestation of *idiopathic* RP is difficult and the interpretation of the literature challenging [84]. Any vessel size can be affected, as supradiaphragmatic vessels more commonly than infradiaphragmatic [85]. SVV can affect the inner ear, eye, or the skin [84]. LVV occurs in approximately 4–18% of idiopathic RP, the thoracic aorta being most commonly affected; MVV usually occurs in addition to LVV, but sometimes only MVV may be present [84–86•]. In any male patient with RP, especially in elderly subjects with a “PAN-like” manifestation (uncommon in idiopathic RP) and macrocytosis, the recently described VEXAS syndrome should be considered [1••, 88••]. SVV and MVV of the CNS are extremely rare [87].

### Rheumatoid Vasculitis in Rheumatoid Arthritis

Rheumatoid vasculitis (RV) has become rare today, most likely due to better disease control [89–91]. It typically appears 10–15 years after the onset of arthritis in patients with erosive disease and high titers of rheumatoid factor or anti-cyclic citrullinated peptide (anti-CCP) antibodies; rarely it antedates arthritis. RV can occur without clinical activity of the arthritis itself but often with extraarticular disease like nodulosis, Felty-syndrome, or rheumatoid eye disease [89, 91]. While LVV does occur, RV typically involves medium-sized and small vessels and can manifest in a “PAN-like” fashion without microaneurysms. Coronaritis and aortitis occur extremely rarely, often diagnosed postmortem [92••]. CNS vasculitis is also very rare and needs to be differentiated from rheumatoid meningitis [91].

### Spondyloarthritis

Aortitis in spondyloarthritis is rare and is described less frequently today than before the advent of biologics [94, 95•]. Histologically, lymphoplasmocytic infiltrates and fibrosis are typical, with the fibrosis being particularly pronounced in the region of the aortic sinuses and aortic valve with possible involvement of the mitral valve and the interventricular septum [92••, 95•]. Several cases of reactive or psoriasis arthritis with similar manifestations were published, but ankylosing spondylitis is the most common subtype associated with LVV [94, 95•]. MVV has not been described in patients with spondyloarthritis; for the few cases with coronary aneurysms, it remains unclear whether they were caused by vasculitis [94].

## Fibroinflammatory Diseases

In these disorders, excessive fibrosis dominates the histological picture and it is accompanied by chronic inflammation. IgG4-related disease (IgG4-RD) can be considered as *the* classical fibroinflammatory disease.

### IgG4-Related Disease

The concept of IgG4-RD exists since approximately 2003. Its typical presentation is insidious; acute or highly inflammatory manifestations usually do not occur [96]. It can be a focal, multifocal, or systemic disease and typically leads to fibrosis and mass effect in the affected organs [96–98]. The involvement of veins alone is rare, but arterial involvement is common; the aorta is usually affected alone or in addition to other vessels in approx. 10–35% of cases. Primary affection of the vessel wall itself (mainly adventitia) occurs less frequently than secondary involvement with perivascular disease (typically periaortitis/retroperitoneal fibrosis). Inflammation of the vessel wall is associated with a tendency to aneurysm formation and possible dissection and rupture whereas periaortal inflammation less frequently leads to luminal changes [96–98]. Vessel stenosis can also occur due to encasing lesions (e.g., superior vena cava, pulmonary or coronary arteries) [97, 98, 127•]. Single cases with cerebral MVV with stenosis or dilatation were reported [128, 129].

### Chronic Periaortitis

Retroperitoneal fibrosis (RPF) is characterized by chronic lymphoplasmocellular infiltrate and fibrosis of retroperitoneal structures with typical ureteral obstruction; it includes idiopathic (75%) and “secondary” forms (25%: malignancies, infections, drugs, carcinoid syndrome, AAV, SLE, Erdheim-Chester etc.) [103•]. Since the disease process typically develops in the adventitia, the majority of idiopathic RPF are located around large arteries (abdominal aorta/iliac arteries > thoracic aorta), where the term chronic periaortitis (CP) is most appropriate [103•, 104]. CP can be subdivided into the entities “periaortic RPF” (no aneurysm; ureteral involvement), “inflammatory abdominal aortic aneurysm” (no ureteral involvement), and “perianeurysmal retroperitoneal fibrosis” (with ureteral involvement) [103•]. The idiopathic forms are difficult to differentiate from IgG4-RD without a biopsy, which usually is only performed if there is evidence for a neoplastic/infectious process or an atypical localization (i.e., not peri-arterial) [101, 103•]. Affection of visceral medium-sized vessel can occur [103•, 104].

## Miscellaneous Inflammatory Diseases

### Sarcoidosis

Sarcoid vasculitis can manifest at any time during the disease. It can involve any vessel size—arteries and veins alike—especially the aorta and the pulmonary vessels (possibly with secondary pulmonary hypertension) [105, 106]. It can also manifest as “PAN-like” MVV, including development of aneurysms, or SVV, especially of the skin, peripheral nerves, and lungs [105, 108, 130]. “TAK-like” LVVs are described, and because granulomatous inflammation is present in TAK and sarcoidosis, it is difficult to differentiate whether the two diseases are concomitantly present or only sarcoidosis [92••, 107, 110]. With the use of vessel-wall imaging, CNS vasculitis is more commonly identified: small perforating arteries and periventricular veins are most often affected, but occasionally also medium-sized cerebral arteries or dural sinuses [109•].

### Thrombangiitis Obliterans or Buerger’s Disease

This manifests as inflammatory vasculopathy of medium and small arteries and veins with typical corkscrew collaterals. Because of the main histologic feature, i.e., a highly cellular intraluminal thrombus with comparatively little involvement of the vessel wall itself, and because the therapy is not immunosuppressive, TO is clearly distinct from other types of vasculitis. Thrombangiitis obliterans (TO) is only exceptionally found in non-smokers and it typically affects infrapopliteal and infrabrachial arteries and superficial veins, but other medium-sized vessels can be involved [112–116].

### Juvenile Temporal Arteritis

This very rare SOV has an imprecise definition. It is more common in males and manifests < 40–50 years of age with a non-tender or painful swelling in the temples, headache (50%), mild eosinophilia (30%), and generally without systemic symptoms or CRP elevation. Histology shows in 90% of cases an eosinophilic infiltrate with perivascular expansion and occasionally some giant cells. There are no known complications or progression to systemic disease. Kimura disease or angiolymphoid hyperplasia with eosinophilia has overlapping features (especially perivascular germinal centers); a clear differentiation from juvenile temporal arteritis can be difficult [131]. IgG4-RD and AAV can demonstrate eosinophil-rich infiltrates of the TA and should be considered.

### Isolated aortitis

This by definition is an SOV, but likely a group of disorders with the same clinical manifestation rather than a common pathophysiology [2, 132]. Isolated aortitis can be found incidentally with the use of cross-sectional imaging (often in the diagnostic process of fever or inflammation of unknown origin) or on histopathology after surgical resection due to aneurysmal dilation or dissection (often without elevation of acute phase reactants prior to the operation) [92••, 132]. Before aortitis can be classified as “isolated,” a comprehensive workup regarding the many diseases that can cause aortitis is advisable, including an FDG-PET/CT if possible. The main differential diagnosis is GCA [133].

## Vasculitis Mimics and Important Other Disorders

It is mostly the similarity of imaging findings (especially stenoses on angiography) and sometimes the clinical manifestations, but much less the histopathological presentation that can mimic vasculitis. The grouping of mimics into partially overlapping categories of vascular manifestations, as well as the size of the affected vessels, should be helpful in compiling a list of differential diagnoses for the individual patient (Table 2). A few selected mimics are discussed individually below. A detailed discussion about vasculitis mimics can be found elsewhere [134•, 135].

### Transient Perivascular Inflammation of the Carotid Artery (TIPIC) Syndrome

This is a rare entity of unknown etiology affecting adults. Presentations is with acute unilateral anterior neck pain with an enhancing periadventitial soft tissue alteration of the carotid bifurcation in 100% and a soft intimal plaque in approximately 50% [136]. Histology can show inflammation and fibrosis [137]. Rarely mild fever, myalgias, dysesthesia, or paresthesia can occur. CRP is rarely mildly elevated. Spontaneously or with the use of nonsteroidal anti-inflammatory drugs, symptoms resolve within a couple of weeks, but slight perivascular thickening persists for months, and relapses can occur [136]. Before transient perivascular inflammation of the carotid artery (TIPIC) is diagnosed, arterial dissection needs to be considered. The persistence of symptoms or marked CRP elevation should raise the suspicion of another disease.

### Erdheim-Chester Disease

Erdheim-Chester disease (ECD) is a rare histiocytosis with a *BRAF-V600E* mutation in approximately 50%. Diagnosis can be delayed for many years due to its insidious onset. Infiltration of multiple organs is typical: bone (> 95%; osteosclerotic lesions, pain in 50%), pituitary (diabetes insipidus, hypogonadism), orbital (exophthalmos), retroperitoneal (“hairy kidney”), CNS (25–50%; pachymeningitis, parenchymal lesions), skin (xanthelasma), or heart (pseudotumor) [138]. Adventitial and periadventitial infiltration leads to its characteristic “coated” aspect. Typically, the aorta but potentially all its large- and medium-sized branches and pulmonary, femoro-popliteal, basilar, and middle cerebral arteries can be affected. Stenosis usually occurs only in medium-sized arteries (e.g., coronary, renal, mesenteric), and vessel dilatation is very rare [139].

### Acute aortic Pathology

LVV can rarely present with chest pain, with consequent suspicion of acute coronary or aortic syndromes. Also, imaging findings of aortic intramural hematoma and dissection can sometimes mimic LVV and vice versa [140, 141].

### Radiation-Induced Vascular Disease

Radiation typically results in inhomogeneous and calcified atherosclerotic changes especially of large arteries, including the aorta, and is limited to the field of radiation. Because the wall alterations can be relatively long, smooth, and concentric, they can mimic LVV [142].

## Conclusion

Considering the multitude of possible etiologies of MVV and LVV as well as their mimics discussed in the present article and other reviews, the correct diagnosis remains a challenge even for experts [1••, 2]. As new entities are constantly described, the astute clinician will reevaluate the diagnosis of any MVV or LVV from time to time, especially in atypical cases [88••]. While it is now common practice to omit biopsy if imaging findings are compatible with MVV or LVV, caution is advised if this approach is used in patients with low to medium pretest-probabilities, because multiple vasculitides and mimics can show similar findings [143]. A thorough clinical examination, medical history and laboratory examination of each patient is mandatory and guides the generation of a differential diagnosis, the choice of imaging modality and whether a biopsy is needed. We clearly advise investing a lot of resources early in the diagnostic process, as the diagnostic accuracy of laboratory tests and imaging rapidly decreases once therapy is started. The diagnosis often remains unclear if investigations were initially performed too superficially. However, even with the most detailed evaluation, there will be situations where an exact entity cannot be identified. Isolated aortitis without the possibility to biopsy is an example.

Regarding the unsatisfactory definitions of vessel sizes, a more precise, consensus-based, and universally accepted definition is desirable. We would be inclined to limit the term “small vessel” to capillaries, venules, and arterioles, since the disease processes and clinical manifestations are rather clearly defined (pulmonary hemorrhage, glomerulonephritis, diffuse purpura of the skin etc.) and the delineation of small muscular arteries from medium-sized muscular arteries seems an impossible task. Defining a “large vessel” is even more difficult but could be approached with a minimum diameter of the artery (i.e., 5 mm), an exhaustive list or by abandoning this principle altogether by defining a new category of medium to large vessel vasculitis. This would make future research studies more comparable.
